# Induction of remission in chronic urticaria by immunotherapy using immunoglobulin/histamine complex (Histobulin™): a case report

**DOI:** 10.1186/s13223-021-00612-8

**Published:** 2021-11-12

**Authors:** Hyuk Soon Kim, Geunwoong Noh

**Affiliations:** 1 Department of Biomedical Sciences, College of Natural Science and Department of Health Sciences, The Graduate School of Dong-A University, Busan, Korea; 2 Department of Allergy, Allergy and Clinical Immunology Center, Cheju Halla General Hospital, Doreongno 65, Jeju-si, 63127 Jeju-Si Jeju Special Self-Governing Province Korea

**Keywords:** Chronic spontaneous urticaria, Immunoglobulin/histamine complex, Histobulin™, Remission, Immunotherapy

## Abstract

**Background:**

Symptom control is a major concern in chronic urticaria. Histobulin™ is a histamine/immunoglobulin complex that has been approved for allergic rhinitis, bronchial asthma and chronic urticaria in some countries. Not only has the immunoglobulin/histamine complex been reported to be effective in allergic diseases, including chronic urticaria, but recently, the possibility of remission induction in chronic urticaria by the immunoglobulin/histamine complex has been reported.

**Case presentation:**

Histobulin™ was administered until remission was induced instead of fixing the number of administrations in four cases of chronic urticaria. Two patients showed an early response and finished treatment with 12 injections of Histobulin™, and the other two patients showed a late response and were injected 43 and 46 times. Remission was induced successfully in all four cases.

**Conclusions:**

Histobulin™ is not only effective but also induces remission in CSU. The Histobulin™ therapy protocol in CSU may be better if the treatment is continued until remission is achieved. Based on the responses of the patients, early responders and late responders were present. The progression of the disease during treatment consisted of a slow improvement phase and a rapid improvement phase. Uniquely, the appropriate allergy laboratory results, including blood eosinophil fraction, total IgE and eosinophil cationic protein level, were normal in all 4 cases. Further studies concerning the mechanisms of Histobulin™ may be needed.

## Background

Urticaria occurs in 0.5–5% of the general population [1]. Chronic urticaria (CU) is diagnosed when the episodes of urticaria last longer than 6 weeks [2]. Chronic urticaria is classified as chronic inducible urticaria (CIU) and chronic spontaneous urticaria (CSU). Histamine, which is released from mast cells, plays a major role in the pathogenesis of CU. The binding of FcεRI and IgE subsequently induces the degranulation of mast cells and the secretion of histamine and leukotriene. Currently, the treatment of chronic urticaria is focused mainly on effective symptomatic control and includes antihistamines, which are used for primary treatment. The disease is not controlled by antihistamines in many cases, and more advanced treatment is needed [2, 3]. Recently, omalizumab, which is a monoclonal antibody to IgE that inhibits the binding of FcεRI and IgE, has been used as a very effective therapeutic [4]. However, despite its effectiveness, recurrence of CSU is well documented with omalizumab treatment [5].

TNF-α inhibitors and intravenous immune globulin (IVIG) are also known to be effective therapeutics [6, 7]. However, these two drugs are not curative therapeutics and have a high cost and/or severe side effects. There is no causative treatment for CSU. The itching and skin lesions in CSU negatively affect the quality of life of patients [8]. Therapeutics for causative treatment in CSU, as well as effective treatment for refractory CSU, are needed.

Histobulin™ (Green Cross PD, Korea) is a histamine-fixed immunoglobulin preparation comprised of 0.15 μg of histamine dihydrochloride and 12 mg of IgG [9], which is approved in chronic urticaria, as well as allergic rhinitis and bronchial asthma, in some countries. Histobulin™ was originally developed from histamine-fixed serum [10], and it can inhibit antigen-induced histamine release from human peripheral blood basophils and rat peritoneal mast cells [11]. Histobulin is known to be effective in allergic rhinitis, bronchial asthma, and atopic dermatitis [12–15].

Immunoglobulin/histamine complexes have been reported to be effective in chronic urticaria for several decades without sufficient reports [16–18]. In a recent report, some patients showed remission, and the remission of chronic urticaria by Histobulin™ treatment was the focus [17]. In this report, Histobulin™ therapy was continued until remission was induced, which is a new therapeutic approach. Finally, remission was induced in all four cases, and two types of clinical courses are described in this report.

### Case presentation

#### Case 1

A 69-year-old Korean female patient visited the Department of Allergy and Clinical Immunology, Cheju Halla General Hospital, due to urticaria and itching for 5 months. She had no specific family history or past medical history. She had been taking fexofenadine daily for 2 months before the visit to our clinic (Table 1). There were no inducible factors, including stress, exercise, cold or sunlight exposure. Based on the diagnostic criteria for CSU, a diagnosis of chronic spontaneous urticaria (CSU) was made [2]. The clinical severity of chronic spontaneous urticaria was evaluated using the Urticaria Severity Score by Jariwala et al. [19]. Of the 12 questions, 10 were scored on a scale of 0–7, with higher scores reflecting greater severity of symptoms, increased disruption of quality-of-life components including sleeping, work/school attendance, and social life, and increased amounts of medication of antihistamine or oral corticosteroid use. Of the 12 questions, 2 reflected body areas experiencing symptoms and had 8 answer choices per question; increased scores suggested more extensive urticaria involvement. One of the USS questions is about the amount of oral corticosteroid used, which was double weighted, and the item score was multiplied by 2 considering that the need for oral corticosteroids reflected an increased severity of disease. However, steroid was not used in this report. The USS could be calculated by adding the score of each question, thereby resulting in a maximum of 93 and minimum of 0.

**Table 1:**
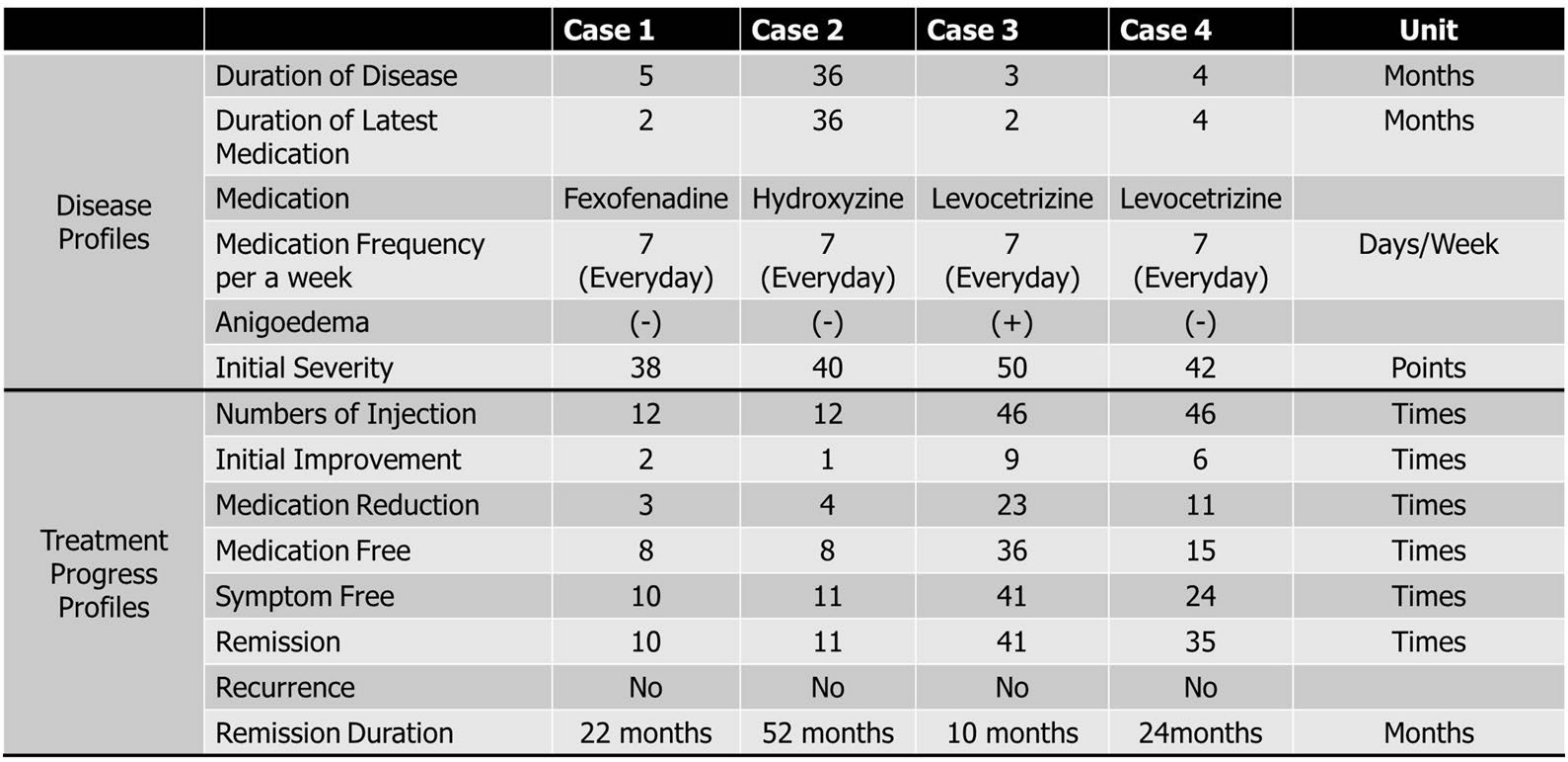
The clinical profiles of disease and treatment progress

The clinical progress of Histobulin^TM^ therapy was characterized as five steps: initial improvement (initial improvement), reduction in medication frequency per week (medication reduction), cessation of medication (medication free), free from the symptoms and signs of CSU, free of symptoms without medication (symptom free) and persistence of symptom-free status for more than 4 weeks without any medication or treatment (remission)

In the clinical severity scoring, the patient scored a 5 and showed initial improvement, the reduction of medication (medication reduction), the state of medication free (medication free), the state of symptom-free (symptom free) and remission (continuous symptom-free more than 4 weeks).

To evaluate allergic status and allergen sensitization for exogenous allergens, basic allergic tests (blood tests and the skin prick test) were conducted before and after treatment. Before the tests, all patients ceased antihistamines for at least 5 days. The patient had blood tests for complete blood counts with differentials, serum eosinophil cationic proteins, serum total IgE and IgE levels for specific allergens using a multiple allergosorbent test (MAST, Green Cross PD, Korea). In the MAST test, the specific IgEs for 41 allergens were evaluated as described in a previous report [20]. The test results showed the levels of specific IgE for each allergen, and a normal negative range was 0.000–0.349 IU/mL. Additionally, a skin prick test was also performed for 53 allergens as described previously [20]. Histamine hydrochloride 10 mg/ml was used as a positive control, and physiological saline was used as a negative control. The allergy results were interpreted by measuring the wheal size. Reactions were read after 15 min and described as negative (0, no reaction), 1 + (reaction greater than the control reaction but smaller than half the size of the histamine reaction), 2 + (equal to or more than half the size of the histamine reaction), 3 + (equal to or more than the size of the histamine reaction) and 4 + (equal to or more than twice the size of the histamine reaction). The minimum size of a positive reaction was 3 mm.

All four patients underwent laboratory tests and skin prick tests before and after treatment. Characteristically, no patients showed abnormally elevated results in the classical allergy tests, including eosinophil fraction in white blood cell counts, basophil fraction in white blood cell counts, eosinophil cationic protein (ECP) and serum total IgE levels (Table 2). However, the sensitization patterns in blood and skin were variable and different among the four cases (Table 3).

**Table 2:**
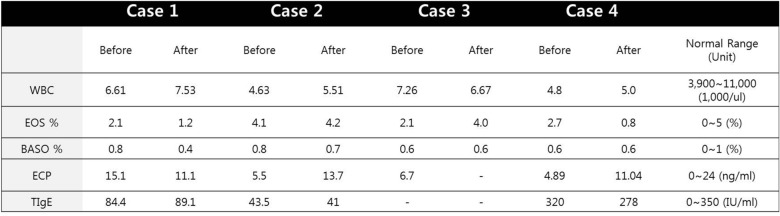
Basic profiles of representative allergic laboratory results, including blood eosinophil and basophil fractions, serum total IgE levels, and serum eosinophil cationic protein levels

**Table 3:**
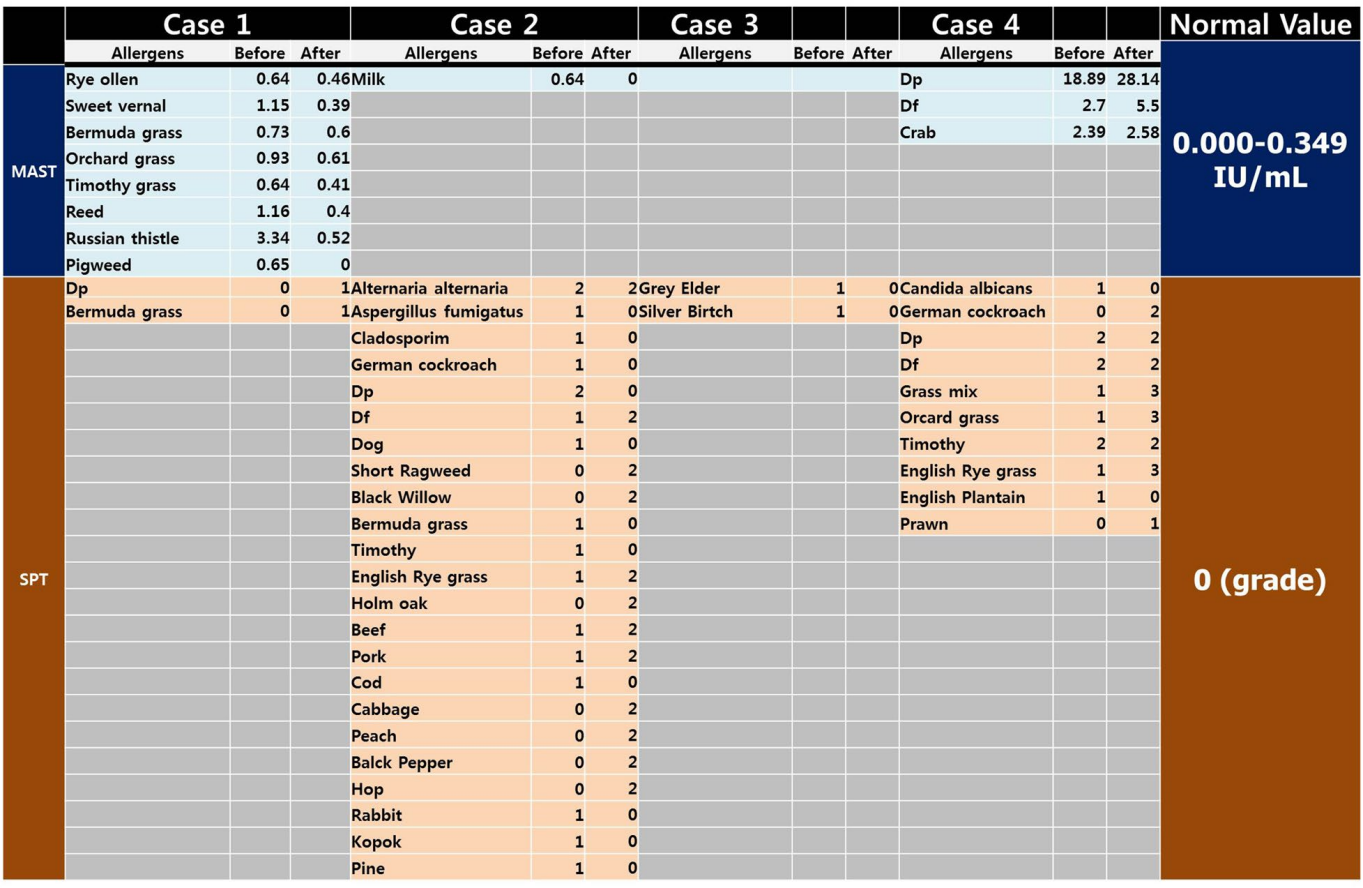
Sensitization profiles to exogenous allergens by a multiple allergosorbent test (MAST, Green Cross PD, Korea) and the skin prick test (SPT)

For MAST, the test results show the level of specific IgE for each allergen, and a normal negative range is 0.000–0.349 IU/mL. SPT was described as negative (0, no reaction), 1+ (reaction greater than control reaction but smaller than half the size of histamine), 2+ (equal to or more than half the size of histamine), 3+ (equal to or more than the size of histamine) and 4+ (equal to or more than twice the size of histamine). The minimum size of a positive reaction is 3 mm

Histobulin™ (Green Cross, Korea) is composed of 12 mg human immunoglobulin/0.15 μg histamine complex (2 ml in an ample). Patients who wanted to cease medication due to CSU participated, and Histobulin™ therapy was conducted to determine whether it induced remission or not, resultantly ceasing medication without symptoms and signs. Histobulin was administered by subcutaneous injection in the deltoid areas of the upper arm every week. In all cases, patients were instructed to take levocetirizine when they were uncomfortable or if the condition interfered with normal living, working or sleeping.

**Fig. 1:**
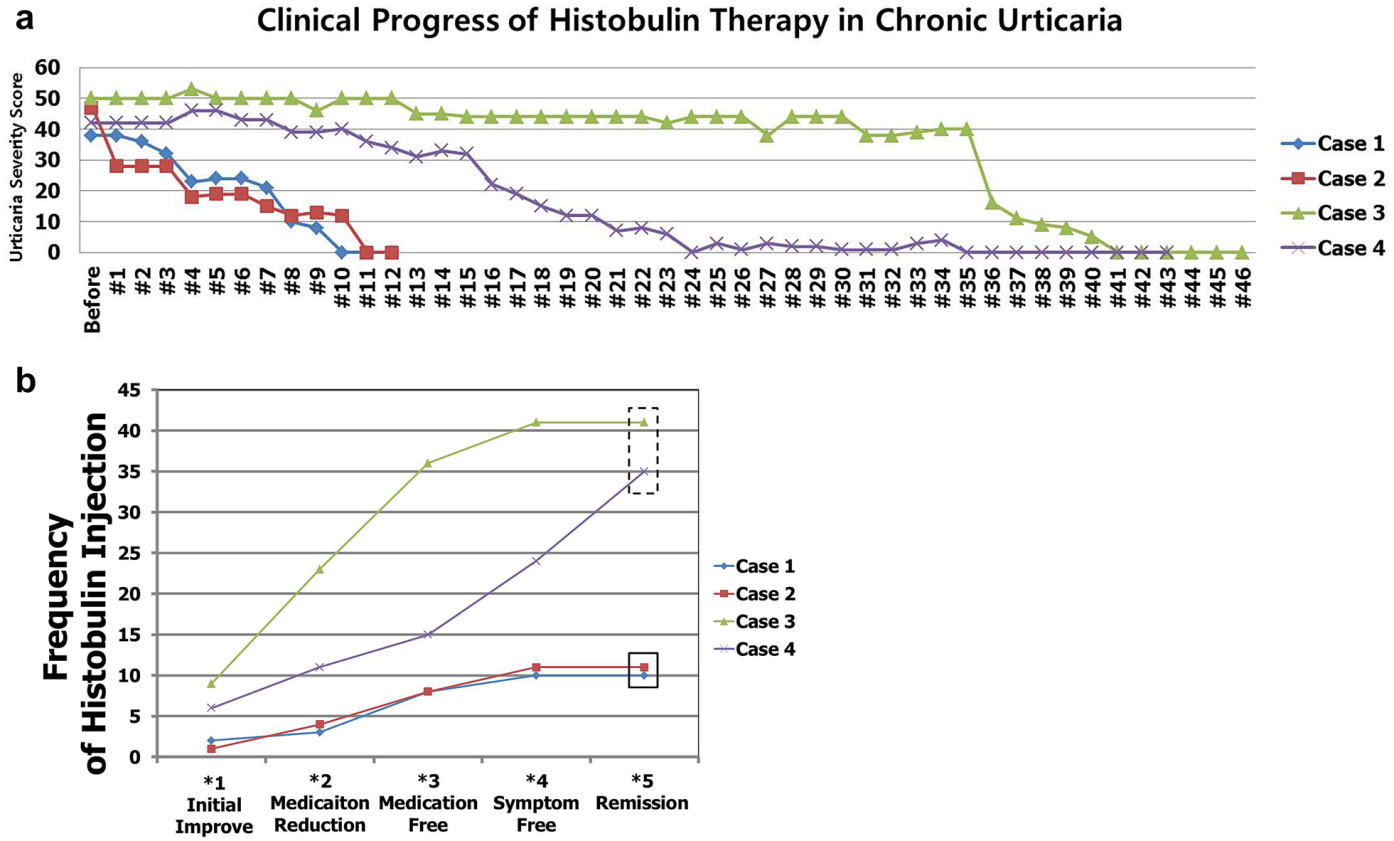
The clinical progress of Histobulin™ therapy in CSU. **a** Clinical progress. **b** Five steps of clinical progress in Histobulin™ therapy: initial improvement (initial improvement), reduction in medication frequency per week (medication reduction), cessation of medication (medication free) without the symptoms and signs of CSU, free of symptoms without medication (symptom free) and persistence of symptom-free status for more than 4 weeks without any medication or treatment (remission)

The patient in case 1 received 12 injections of Histobulin. Her initial clinical severity was 38 points. The clinical progress is shown in Fig. 1a. She showed initial clinical improvement after the second injection (Initial Improvement) (Fig. 1b; Tables 1, 2, 3). The weekly medication frequency was reduced after the third injection (Medication Reduction). After the eighth injection, she no longer took medication (Medication Free). She was symptom free (Symptom Free) continuously (Remission) after the tenth injection. Remission was defined when symptoms and signs were not present for 4 weeks without medication. Her remitted state was maintained for more than 18 months, i.e., until the present.

#### Case 2

A 63-year-old Korean female patient visited the Department of Allergy and Clinical Immunology, Cheju Halla General Hospital, due to urticaria and itching for 3 years. She had no specific family history or past medical history. There was no specific inducible factor for the development of urticaria. She had been taking hydroxyzine every other day for 3 years before visiting our clinic (Table 1). She met the diagnosis of CSU.

Histobulin was injected subcutaneously every week while the patient took levocetirizine if necessary, as described in case 1. Histobulin™ was administered to her 12 times. Her initial clinical severity was 40 points (Fig. 1a). She showed an initial clinical improvement after the first injection (Fig. 1b, Table 1). Weekly medication frequency was reduced after the fourth injection (Medication Reduction). After the eighth injection, she no longer took medication (Medication Free). She was symptom free (symptom free) continuously (remission) after the eleventh injection. Her remission has been maintained for more than 4 years, i.e., until the present.

#### Case 3

A 53-year-old Korean female patient visited the Department of Allergy and Clinical Immunology, Cheju Halla General Hospital, due to urticaria, itching and angioedema on the face every 15 days for 3 months. Urticaria and itching developed, and angioedema followed. The symptoms and signs persisted for 7 days. Recently, she took levocetirizine every other day for 2 months (Table 1). She had no specific family history. In her past medical history, she was diagnosed with colon cancer and received surgical treatment 6 months prior. There was no specific inducible factor for urticaria, and her diagnosis was CSU.

Histobulin was injected subcutaneously every week while the patient took levocetirizine if necessary, as described in case 1. Histobulin was given 46 times. Her initial clinical severity was 50 points (Fig. 1a). She showed an initial clinical improvement after the ninth injection (initial improvement) (Fig. 1b; Table 1). The weekly medication frequency was reduced after the twenty-third injection (Medication Reduction). After the thirty-sixth injection, she no longer took medication (Medication Free). She was symptom free (symptom free) continuously (remission) after the 41st injection. Her remission has been maintained for more than 6 months, i.e., until the present.

#### Case 4

A 51-year-old Korean female patient visited the Department of Allergy and Clinical Immunology, Cheju Halla General Hospital, due to urticaria and itching for 4 months. She was taking levocetirizine every other day for 4 months (Table 1). She had no specific family history or past medical history. She had no specific inducible factor for the development of urticaria. Her diagnosis was CSU.

Histobulin was injected subcutaneously every week while the patient took levocetirizine if necessary, as described in case 1. Histobulin was given 46 times. Her initial clinical severity was 42 points (Fig. 1a). She showed an initial clinical improvement after the sixth injection (Fig. 1b; Table 1). Medication frequency per week was reduced after the eleventh injection (Medication Reduction). After the fifteenth injection, she took medication no more (Medication Free). Her symptoms and signs were no longer present (symptom free) after the twenty-fourth injection, and her symptom-free status was maintained continuously (remission) after the thirty-fifth injection. Her remission has been maintained for more than 24 months, i.e., until the present.

## Discussion and conclusions

### Effectiveness of Histobulin™

The major points of this report are that remission was achieved by Histobulin™ in CSU and that Histobulin™ was effective. Despite the results that remission was observed in some patients in a previous report [17], in this trial, a new concept was applied, which consisted of continuing Histobulin™ treatment until remission was induced, rather than fixing the treatment frequency and period. The frequency of injection that led to the actual clinical improvement in one patient was 30 times the number of injections in another (Table 1; Fig. 1b). A previous report found that Histobulin™ therapy may be ineffective with only 12 injections [11]. Considering that symptomatic control is currently the major target of treatment for CSU in international guidelines [2], the induction of remission with Histobulin™ is a revolutionary result.

Omalizumab is used for refractory chronic urticaria [4], but it is not a curative treatment. A high cost and the need for repetitive treatments due to recurrences [5] are limitations of the treatment. If Histobulin™ induces remission, it might be better than omalizumab therapy, which is considered an effective therapeutic in CSU. Histobulin™ therapy is indicated in several situations, including cases in which, due to quality of life issues, patients want to be relieved of chronic urticaria symptoms and cease medication.

### Laboratory characteristics of chronic spontaneous urticaria

**Fig. 2:**
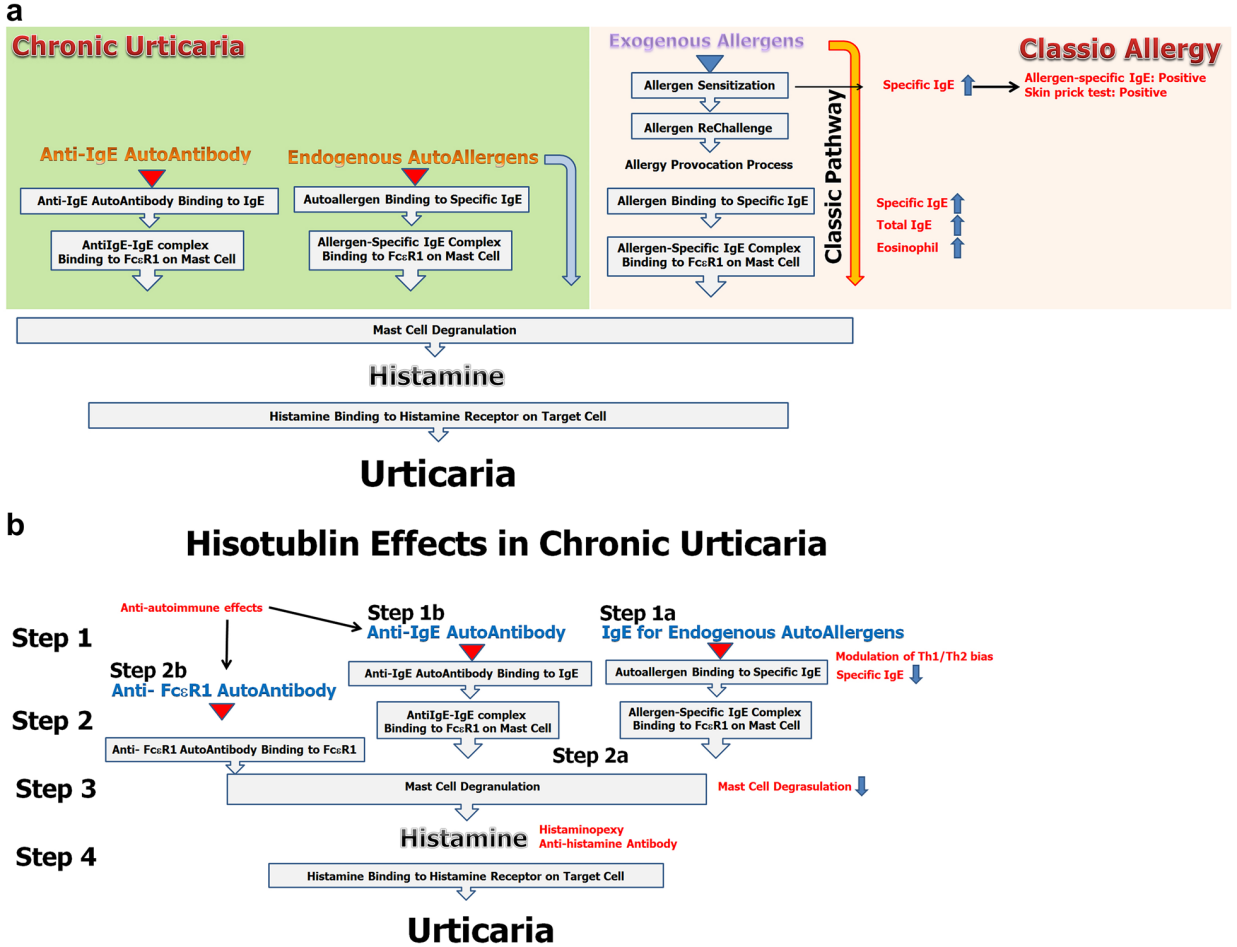
**a** Laboratory characteristics in CSU. In the classic allergic pathway, allergy sensitization and provocation by exogenous allergens and subsequent mast cell granulation and allergen-specific Th2 activation result in an increase in allergen-specific IgE (SIgE), serum total IgE (Total IgE), blood eosinophil fraction and serum eosinophil cationic protein. However, the immunopathogenesis of CSU bypasses the exogenous allergen-IgE-FcεRI pathway, and the representative allergy laboratory tests were possibly all negative. **b** Revised action mechanisms of Histobulin™ in CSU. The immunopathogenesis of CSU was revised step by step as autoantigen-specific IgE and autoantigen binding (step 1a), the IgG or IgM autoantibody binding to IgE (step 1b), binding of autoantigen-IgE complex or anti-IgE IgG/IgM antibody-IgE complex to FcεR1 (step 2a), binding of anti-FcεR1 autoantibody to FcεR1 (step 2b), histamine release with mast cell degranulation (step 3) and high level of histamine and binding to histamine receptor which leads to clinical manifestations of allergy (step 4). Histobulin™ has the effects of histaminopexy and induces antihistamine antibodies. Histobulin™ possibly decreases serum histamine levels at step 4. Histobulin™ inhibits antigen-induced histamine release from human peripheral blood basophils at step 3. Histobulin™ decreases specific IgE clinically and affects step 1a (IgE antibody to autoantigens). The main constituent of Histobulin™ is immunoglobulin, and IVIG effects were possibly expected in Histobulin™. Histobulin™ may be effective in step 1b (IgG and IgM autoantibody to IgE) and step 2b and step 2b (autoantibody to FcεR1)

The main pathogenesis of CSU is mast cell degranulation and histamine release, which are common in allergic diseases. In the classic pathway, allergy sensitization and provocation by exogenous allergens and subsequent mast cell granulation and allergen-specific Th2 activation result in Th2 cytokine production, including IL-4 and IL-5 production. Consequently, serum total IgE, allergen-specific IgE and blood basophil fraction are increased by IL-4 through repetitive allergen challenges. In particular, in atopic dermatitis, the blood eosinophil fraction and serum eosinophil cationic protein were increased by IL-5 through repetitive allergenic challenges. Therefore, representative allergy laboratory tests, including blood eosinophil and basophil fractions, serum eosinophil cationic protein, and serum total IgE levels, are possibly increased in allergic diseases. However, the immunopathogenesis of CSU does not follow the classic pathway bypassing the exogenous allergen-IgE-FcεRI pathway (Fig. 2a), and the representative allergy laboratory tests were likely all negative. In the four patients examined in this report, the laboratory results were all negative (Table 2).

Exogenous allergens are not causes of CSU, and at least in some cases, the sensitization profile in the serum allergen-specific IgE test and skin prick test may be negative, or their results may not be significant clinically. However, itching possibly changes the lesion site as Th2 polarization occurs through TSLP and IL-21 [21]. In this situation, allergen sensitization to exogenous allergens might occur. Due to itching and urticaria, patients may scrape the skin, and invading allergens can be introduced. Allergen sensitization can occur in these conditions as it does in normal subjects without any allergic disease. Therefore, allergen sensitization profiles in the blood allergen-specific IgE test and the skin prick test will be positive for allergens, although the positive allergens are not directly related to chronic urticaria. Conclusively, all the allergy laboratory tests and the allergen sensitization profiles are conceptually negative in CSU. However, allergen sensitization profiles can show positive allergens according to the clinical conditions, as in this report (Table 3).

### Concept of treatment protocol for Histobulin™

The clinical difference of this case report is the concept of the treatment protocol. In the previous treatment using immunoglobulin/histamine complex, the duration of treatment and the numbers of injections were fixed, and the clinical results were evaluated. Fortunately, remission was observed in some patients in another previous report [17], and the possibility that the immunoglobulin/histamine complex induces remission in CSU was suspected. In this study, Histobulin™ treatment continued, and remission was induced in all 4 cases of CSU.

Clinically, the injection numbers until remission were achieved were different among the patients. Two patients responded early, and remission was also induced within 12 injections (Table 1; Fig. 1a, b). The other two cases responded, and remission was induced after more injections, i.e., as many as 41 injections. Therefore, Histobulin™ treatment should be approached not with a fixed number of injections or period of time but by waiting for remission with whatever numbers of injections are required because, according to the patients, the response to Histobulin™ treatment was different.

### Characteristics of clinical progress in Histobulin™ therapy: clinical landmarks

From the results, the clinical progress of Histobulin™ therapy was characterized as five steps. First, the initial improvement (initial improvement) could be observed (Table 1; Fig. 1b). Second, the medication frequency was reduced (medication reduction). Third, patients ceased to take medication at the end (medication free) without being bothered by the symptoms and signs of CSU. Fourth, patients reached symptom-free status for a week during treatment (symptom free) without medication. Fifth, if patients were free of CSU symptoms and signs for more than 4 weeks without any medication or treatment, most achieved remission status for a long time (remission). These four cases have not shown recurrence. When patients showed remission, the treatment was stopped.

Detection of initial improvement is very important because, if patients showed initial improvement clinically, it may be expected that Histobulin™ is effective and patients may achieve remission at the end. The point of the initial improvement was also different among the patients, from 1 to 9 injections. In case 3, the patient felt and described improvement after the ninth injection; namely, the initial improvement was shown 9 weeks after the beginning of treatment. In this case, both the patient and physician wondered if Histobulin™ was effective. In the treatment protocol of 12 injections, this patient might be classified as an ineffective case.

Medication frequency per week was reduced during treatment. The interval of medication was directly related to the medication frequency. After the interval of medication became 6 days, patients did not take any more medication. A medication interval of 7 days or more was not present, and a medication-free status was reached. If the medication interval was 6 days, the physician may have predicted that medication-free status would come. From these results, Histobulin™ also seems to have the effects of symptomatic relief. Among several immunologic actions for the therapeutic effects of Histobulin™, histaminopexy reduces the blood histamine level. Through a mechanism of histaminopexy, Histobulin™ is enough to temporarily improve the clinical symptoms and signs of CSU.

At the beginning of this study, it was questionable whether remission was achieved by Histobulin™ therapy. As a result, in all cases, remission was achieved through the symptom-free stage.

### Two kinds of responders to Histobulin™

Based on the mechanisms of action of Histobulin™, the therapeutic effects of Histobulin™ should be assessed step by step and revised systemically (Fig. 2b). Step 1 is autoantigen-specific IgE and autoantigen binding (step 1a) [22]. Another step 1 is the IgG or IgM autoantibody binding to IgE (step 1b). Step 2 is binding of the autoantigen-IgE complex or anti-IgE IgG/IgM antibody-IgE complex to FcεR1 (step 2a). Another step 2 is binding of the anti-FcεR1 autoantibody to FcεR1 (step 2b) [23]. Step 3 is histamine release with mast cell degranulation. Step 4 is a high level of histamine binding to the histamine receptor, which leads to clinical manifestations of allergies.

Histobulin™ was developed for the effects of histaminopexy and decreases serum histamine levels [10] (Fig. 2b). With histaglobin (immunoglobulin/histamine complexes, compatible with Histobulin™) therapy, the value of histaminopexy rose over 240%, which was well correlated with the clinical effects in chronic urticaria [24]. Moreover, Histobulin™ induced antibodies to histamine [16, 25]. With the effects of histaminopexy and/or antihistamine antibodies, Histobulin™ seems to reduce the histamine level at step 4. Histobulin™ inhibits antigen-induced histamine release from human peripheral blood basophils [11] and seems to be effective in step 3. Recently, Histobulin™ decreased specific IgE clinically [26] and may be effective in step 1a.

Intravenous immune globulin (IVIG) suppresses NFκB activation [27]**,** and Histobulin™ also inhibits NFκB nuclear translocation [28]. IVIG affects Th1/Th2 imbalance [29]**,** and Histobulin™ modulates Th1/Th2 bias [30]. Histobulin™ seems to have the effects of IVIG. The main constituent of Histobulin™ is immunoglobulin, and Histobulin™ seems to have a small quantity of IVIG for subcutaneous use. IVIG effects were possibly expected in Histobulin™. IVIG is effective in autoantibody-mediated autoimmune diseases such as idiopathic thrombocytopenic purpura (ITP), in which anti-platelet antibody is positive [31]. Histobulin™ was reported to possibly be effective in autoimmune diseases [26]. From this viewpoint, Histobulin™ may be effective in step 1a (IgE antibody to autoantigens), step 1b (IgE and IgM autoantibody to IgE) and step 2b (autoantibody to FcεR1). Overall, Histobulin™ seems to be effective in CSU by affecting most of the levels of immunopathogenesis of CSU.

Despite the examination of just 4 cases, there were two kinds of responders found: early responders and late responders. These seem to be related to the mechanisms of action of Histobulin™ and the pathogenesis of CSU. Histamine plays a central role in the clinical manifestations of CSU, including itching and urticarial eruption. The basic effect of Histobulin™ is histaminopexy, which is enough to improve the initial symptoms. The frequencies of injections until patients showed initial improvement were different in the four cases, which may be because the severity of CSU through the effects of histamine also seems to be different among the patients. This may be related to the sensitivity of patients to histamine and/or the level of histamine in the circulation or tissues.

However, the histaminopexy effects of Histobulin™ are inevitably temporary. It is well known that autoimmune mechanisms participate in the pathogenesis of CSU. In this circumstance, without the resolution of these autoimmune mechanisms, remission induction is impossible in CSU. However, long-term remission was induced, and there was no recurrence in any of the four cases. Autoimmune mechanisms in the cases of this report seem to be solved by Histobulin™.

### Two phases of improvement in Histobulin™ therapy

In particular, in the late responders, two phases of clinical courses were observed: the early slow improvement phase and the late abrupt improvement phase. In the early slow improvement phase, patients showed slow or steady or even no change after initial improvement until the 35th injection in case 3 and the 14th injection in case 4 (Fig. 1a). In the late rapid improvement phase, rapid improvement was observed between the 36th and 41st injections in case 3 and between the 15th and 24th injections in case 4.

The main action mechanisms of Histobulin™ were suspected to be different in the early slow improvement phase and late abrupt improvement phase. It is suspected that histaminopexy may theoretically be related to the early slow improvement phase and that the resolution of autoimmune mechanisms may be related to the late rapid improvement phase.

The early responders mainly showed only the rapid improvement phase, which is similar to the curve of the late rapid improvement phase of the late responders in which the autoimmune mechanisms seem to be resolved just after Histobulin™ treatment. From the clinical courses, the immunopathogenesis of CSU related to the rapid improvement phase seems to be the essential point to be solved in the disease for remission induction and causative treatment.

### New concept of allergy: classification of allergic disease

Considering the immunopathogenesis of CSU, basically the general allergic laboratory test and the skin prick test are normal, as the laboratory results in all 4 cases of this report, because CSU occurs via an alternative allergy pathway rather than the classic pathway in which allergic sensitization to exogenous allergens occurs and allergy provocation by sensitized allergens follows (Fig. 2a). From these points, CSU is a histamine-mediated disease with skin manifestations and is nonallergen-specific. Additionally, from a therapeutic perspective, Histobulin™ therapy is a nonallergen-specific immunotherapy. Accordingly, allergies might be classified as allergen-specific allergies and nonallergen-specific allergies.

Antihistamine, steroids and other immunosuppressants, including cyclosporine A and omalizumab, are also nonallergen-specific treatments, but they are temporary symptomatic treatments that mask terminal actions in disease pathogenesis rather than curative or causative treatments.

However, several curative/causative nonallergen-specific treatment agents that improve immunopathogenesis have been reported recently, such as IFN-γ, which results in polydesensitization effects in allergic diseases [32]. Nonallergen-specific polydesensitization effects of Histobulin™ have been reported [26]. Based on the action mechanisms of Histobulin™, it may need to be classified as a nonallergen-specific histamine-mediated disease.

### Clinical trial of immunoglobulin/histamine complex therapy in allergic diseases, including chronic urticaria, and limitations of this study

Since the immunoglobulin/histamine complex was developed by Parrot et al. in 1954 [10] and suggested for the treatment of allergies [33], the immunoglobulin/histamine complex has been tried clinically in several studies. Histaglobin (immunoglobulin/histamine complex, compatible with Histobulin™) has been used in allergic dermatoses, including atopic dermatitis, eczema and neurodermatitis, since the 1960s in Russia, Italy and Japan [13, 34–38]. However, intensive clinical trials, as well as basic immunologic research on the clinical effects and the immunopathogenic action mechanisms of the immunoglobulin/histamine complex, were not continued despite its long history of clinical application. Moreover, the studies were mainly restricted to specific areas, such as some countries in Eastern Europe and Asia, and in India and Russia due to unknown reasons. In 1970, the effects of immunoglobulin/histamine complex therapy on antihistamine factors in chronic relapsing urticaria were reported as “histaglobin” in Russia [39]. Immunoglobulin/histamine complex (Histaglobin) therapy was tried in the treatment of chronic urticaria, as well as occupational dermatoses, in Russia in 1973 [40]. Therapeutic effectiveness was evaluated in 45 patients with allergic rhinitis and chronic urticaria in the study by a Russian allergist, Gushcin et al. in 1999 [16], and after more than 10 years in 51 patients with chronic urticaria, in the study of Indian allergist, Rajesh et al. in 2016 [17]. However, in the study of Rajesh et al., some patients with chronic urticaria were remitted by histaglobulin (immunoglobulin/histobulin complex, compatible with Histobulin™), which became an initiative in our unpublished clinical results for this case study being conducted simultaneously. Thus, we were encouraged to evaluate the possibility of remission induction in chronic urticaria.

CSU has major detrimental effects on the quality of life and causes severe stress [41]. Apart from treatment costs, CU is associated with a high consumption of medical resources and other indirect costs in Europe and the USA, which results in a global burden of chronic urticaria for the patient and society [42]. Until now, international guidelines have focused on the symptomatic control of chronic urticaria step by step [43]. The therapeutic modality for remission induction, as well as the causative treatment in chronic urticaria, is absolutely and urgently necessary. In this situation, alternative treatments for chronic spontaneous urticaria beyond the guideline algorithm were suggested, and in this paper, immunoglobulin/histamine complexes were suggested as alternative therapeutics [18]. This case study was conducted as the first step to evaluate the possibility of remission induction in chronic urticaria using Histobulin^TM^ with a different concept of treatment.

Histobulin^TM^ is much less expense than omalizumab at less than 1/10 the price and has few considerable side effects because its main component is a small dose of immunoglobulin. A limitation of this case report is that the number of cases was too small. Moreover, the duration of illness of three patients was less than 1 year, and they possibly remitted spontaneously. For confirmation of clinical effects, double-blinded placebo-controlled tests with patients on a large scale are necessary.

Conclusively, the results of this case report suggest that Histobulin™ seems not only to be an effective treatment for CSU but may also induce remission. The conceptual prototype of the Histobulin™ therapy protocol in CSU may be to continue the treatment until remission is achieved. According to the patients, there seem to be two types of responses: early responders and late responders. Additionally, the progress of treatment seems to consist of a slow improvement phase and a rapid improvement phase. Uniquely, the representative allergy laboratory results, including blood eosinophil fraction, total IgE and eosinophil cationic protein level, did not show abnormally elevated results, at least in all 4 patients of this case report. To confirm these effects, a large-scale clinical trial of a double-blinded placebo-controlled study and basic studies on the immunologic action mechanisms are absolutely necessary.

## Data Availability

Not applicable.

## Abbreviations

CSU: Chronic spontaneous urticarial
Dp: Dermatophagoides pteronyssinus
Df: Dermatophagoides farina
